# Can SNOMED CT be squeezed without losing its shape?

**DOI:** 10.1186/s13326-016-0101-1

**Published:** 2016-09-21

**Authors:** Pablo López-García, Stefan Schulz

**Affiliations:** Institute for Medical Informatics, Statistics and Documentation, Medical University of Graz, Auenbruggerplatz 2, Graz, Austria

**Keywords:** SNOMED CT, Ontology modularization, Biomedical terminology

## Abstract

**Background:**

In biomedical applications where the size and complexity of SNOMED CT become problematic, using a smaller subset that can act as a reasonable substitute is usually preferred. In a special class of use cases—like ontology-based quality assurance, or when performing scaling experiments for real-time performance—it is essential that modules show a similar shape than SNOMED CT in terms of concept distribution per sub-hierarchy. Exactly how to extract such balanced modules remains unclear, as most previous work on ontology modularization has focused on other problems. In this study, we investigate to what extent extracting balanced modules that preserve the original shape of SNOMED CT is possible, by presenting and evaluating an iterative algorithm.

**Methods:**

We used a graph-traversal modularization approach based on an input signature. To conform to our definition of a balanced module, we implemented an iterative algorithm that carefully bootstraped and dynamically adjusted the signature at each step. We measured the error for each sub-hierarchy and defined convergence as a residual sum of squares <1.

**Results:**

Using 2000 concepts as an initial signature, our algorithm converged after seven iterations and extracted a module 4.7 % the size of SNOMED CT. Seven sub-hierarhies were either over or under-represented within a range of 1–8 %.

**Conclusions:**

Our study shows that balanced modules from large terminologies can be extracted using ontology graph-traversal modularization techniques under certain conditions: that the process is repeated a number of times, the input signature is dynamically adjusted in each iteration, and a moderate under/over-representation of some hierarchies is tolerated. In the case of SNOMED CT, our results conclusively show that it can be squeezed to less than 5 % of its size without any sub-hierarchy losing its shape more than 8 %, which is likely sufficient in most use cases.

## Background

The large size and complexity of SNOMED CT [1] constitute a problem in many biomedical applications and studies have shown that using a much smaller subset of interest is often sufficient [2]. Applications include problem lists [3], tagging medical images [4], and annotating texts from cardiology [5], among others. A well-known example of the benefits of using a subset of interest is the CORE problem list subset of SNOMED CT, which contains only 16 874 terms (roughly 1 % the terms of SNOMED CT), while covering over 95 % of its usage [6].

The theory of how to extract such subsets is studied by the ontology modularization area of research [7]. Ontology modularization techniques are generally focused on obtaining a minimal subset (also called module or segment) that maximally covers a specific domain or that is representative for a particular application. This is the case of the problem lists or annotation cases mentioned above, or the study by Seidenberg and Rector [8], where they described how they extracted a representative segment of the GALEN ontology [9] for cardiology using the seed concept *‘Heart’* as a signature.

A *signature* is an initial set of concepts (called *seeds*) that bootstraps the modularization process, on which many ontology modularization techniques rely, including graph-traversal [8, 10–12] and logic-based techniques [13, 14].

Often, these modules are not *balanced* when it comes to representing the original distribution or shape of sub-hierarchies shown by the original ontology or terminology. For example, in the CORE problem list subset of SNOMED CT, most concepts belong to the *Clinical Finding*, *Procedure*, *Situation with Explicit Context*, and *Event* sub-hierarchies. The opposite case is also possible: in a previous study, we found that modules can excessively and uncontrollably grow and spread across sub-hierarchies, especially when using graph-traversal techniques [5].

These results are not surprising, because most prior work on ontology modularization has not focused on preserving the representativity of the sub-hierarchies of the original ontology, so the shape of the original ontology is inevitably lost in the modules.

There is a special class of use cases, however, where it is essential that modules are representative of the sub-hierarchies of the original ontology and therefore show a similar shape, such as:

- In ontology-based quality assurance, where small but representative samples of a huge ontology are to be inspected [15];
- for obtaining a demonstration version that is understandable for users or facilitates visualization [16, 17];
- for alignment with a highly constrained upper level ontology, such as the Basic Formal Ontology (BFO) [18], especially the upcoming BFO 2.0 OWL version, which includes relations, DOLCE [19] or BioTopLite [20], where reasoning has to be tested on small subsets and in iterative debugging steps;
- for performing scaling experiments for real-time performance of a large OWL DL ontology;
- for the description logics community, who welcomes scalable testbeds for developing tools like editors and reasoners.

To the knowledge of the authors, little research on ontology modularization has focused on extracting balanced modules for such applications, where keeping the original shape of a large ontology such as SNOMED CT in terms of its sub-hierarchies is a requirement.

In this paper, we study the concept distribution of SNOMED CT’s sub-hierarchies, and we propose and evaluate an iterative algorithm for extracting balanced modules. Our main goal is to investigate to what extent it is possible to obtain modules that preserve the original shape of SNOMED CT in order to be used in our identified class of use cases.

## Methods

As input for our experiments, we used the July 2014 International Release of SNOMED CT [21]. We first generated its corresponding OWL-EL version using the Perl script included in SNOMED CT’s official distribution. We then removed the *SNOMED CT Model Components* sub-hierarchy, which contains metadata concepts only. For the remainder of this text, we refer to SNOMED CT and our input version (containing 229 330 classes) termed *SCT* interchangeably.

### SNOMED CT concept distribution

**Fig. 1:**
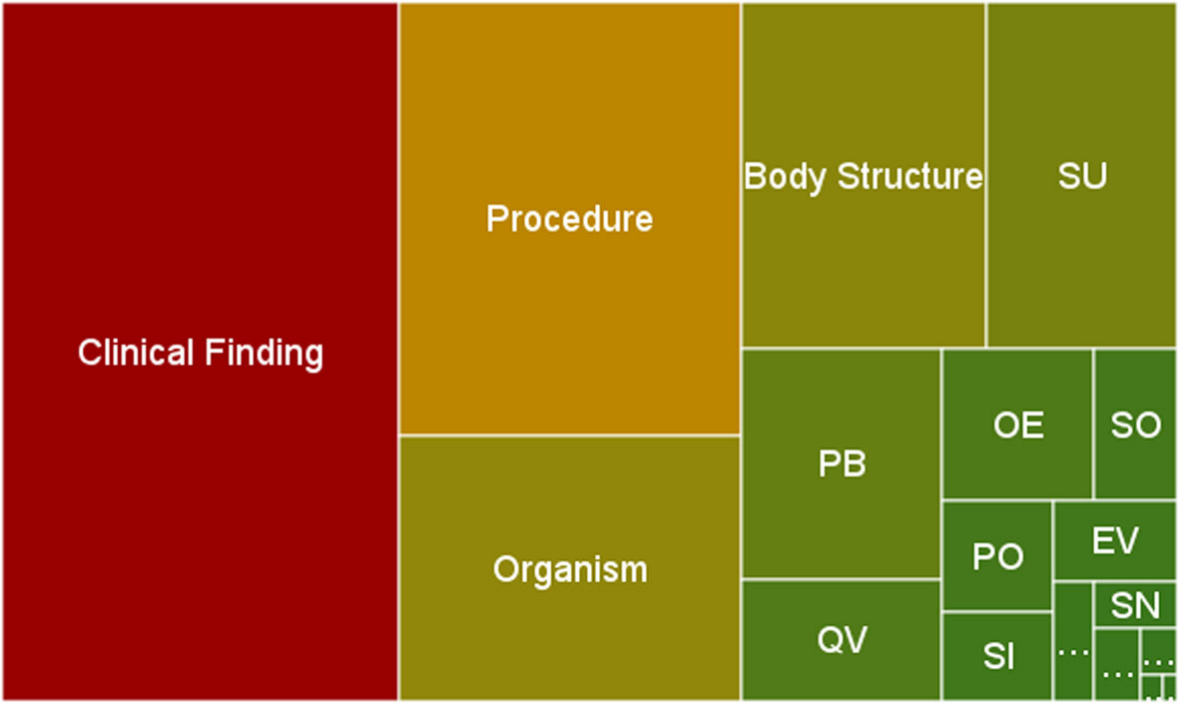
SNOMED CT’s shape represented with a treemap. Sub-hierarchies containing less than 10 % of SNOMED CT concepts are shown in acronyms (see Table 1)

Table 1 shows the main 18 sub-hierarchies of SNOMED CT and their concept distribution. Four sub-hierarchies (*Clinical Finding*, *Procedure*, *Organism*, and *Body Structure*) contain over 10 % of SNOMED CT’s concepts each, accounting for over 70 % of the concepts when considered altogether. As a useful way of visualizing concept distribution and for comparative purposes (see Section “Results”), the same information is displayed in the form of a treemap in Fig. 1. The treemap represents SNOMED CT’s hierarchical information as a set of colored rectangles, where the area (and color) of each rectangle is proportional (and darker/lighter) to the number of concepts in the sub-hierarchy.

**Table 1:** Main sub-hierarchies of SNOMED CT. The metadata concepts sub-hierarchy (*SNOMED CT Model Components*) was not considered

Subhierarchy (Abbreviation)	Concepts	Distribution
Clinical Finding (CF)	100 893	33.57 %
Procedure (PR)	53 914	17.94 %
Organism (OR)	33 273	11.07 %
Body Structure (BS)	30 685	10.21 %
Substance (SU)	24 021	7.99 %
Pharmaceutical/Biologic Product	16 881	5.62 %
Qualifier Value (QV)	9 055	3.01 %
Observable Entity (OE)	8 307	2.76 %
Social Context (SO)	4 703	1.56 %
Physical Object (PO)	4 522	1.50 %
Situation with Explicit Context (SI)	3 695	1.23 %
Event (EV)	3 673	1.22 %
Environment or Geogr. Location (EG)	1 814	0.60 %
Specimen (SN)	1 447	0.48 %
Staging and Scales (ST)	1 309	0.44 %
Special concept (SP)	649	0.44 %
Record Artifact (RA)	227	0.22 %
Physical Force (PF)	171	0.08 %

### Balanced SNOMED CT modules

In a comprehensive study, d’Aquin et al. [22] concluded that there is no universal way to extract ontology modules and that the chosen approach should be guided by each domain or application. It is therefore important to clearly define what constitutes a module. For our purposes, presented in the introduction, we define a *balanced SNOMED CT module* (*M*) as a minimal collection of classes from *SCT* that conform to the following requirements:

- All classes in *M* are hierarchically connected to SNOMED CT’s root concept in the same way as in *SCT*.
- All classes in *M* share the same axiomatical class definition as in *SCT*.
- Sub-hierarchies in *M* are distributed (approximately) in the same proportion as in *SCT*. In practical terms, when visualized using a treemap, *M* should look similar to the treemap of SNOMED CT shown in Fig. 1.
- Our model is restricted to classes. SNOMED CT metadata concepts are excluded and not subject to modularization.

### Module construction from seeds

**Fig. 2:**
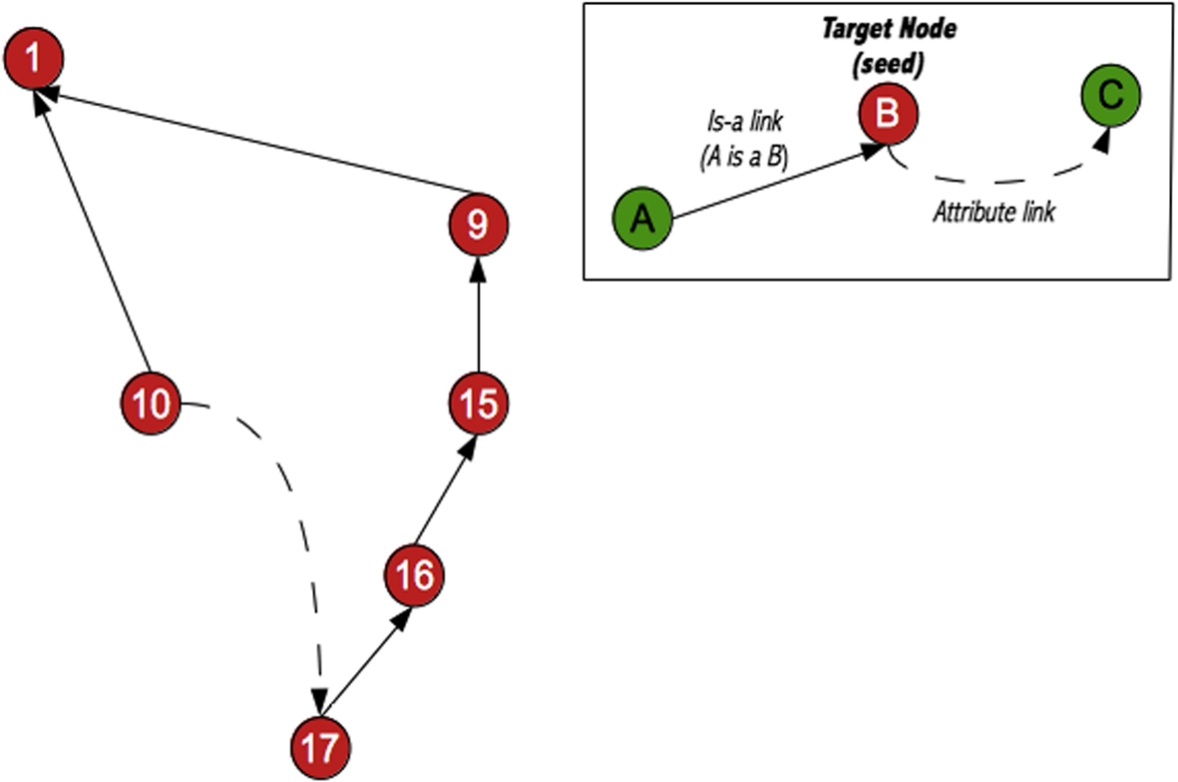
Ontology modularization strategy to build our module *M*, starting from the seed concept (target node) labeled as ’10’. Figure 3 shows the original ontology from which it was extracted

**Fig. 3:**
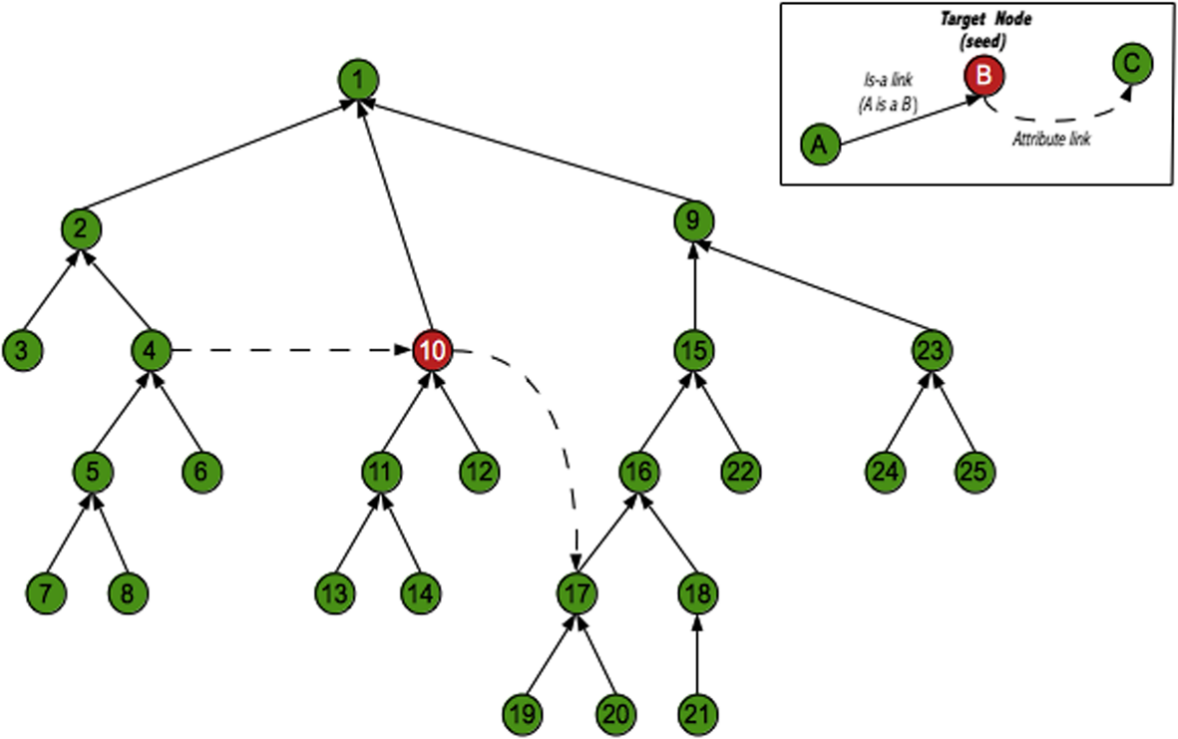
Sample ontology, with an initial signature containing the seed concept (target node) labeled as ’10’

To extract our module *M*, we followed a graph-traversal ontology modularization approach, adapted from Seidenberg and Rector [8]. Using their terminology, concepts (in our case, classes) are represented as nodes in a graph, and seed concepts are called *target nodes*. The strategy takes seeds that conform an initial signature as input, and then iteratively adds classes that appear in the right-hand expressions of their definitions (i.e., are connected by attribute links) and their links up the hierarchy, then becoming new target nodes. Figure 2 shows an example of a resulting module, where it can be seen that (a) all classes are hierarchically connected to the root concept in the same way as in the original ontology (Fig. 3), and (b) all classes share the same axiomatical class definition as the original ontology (i.e., show the same structure when displayed as a graph).

### Seed adjustment: an iterative algorithm

The strategy to build a module using seeds presented above guarantees requirements (a) and (b) from our definition of *M*, but does not guarantee requirement (c), i.e., that sub-hierarchies in *M* will be distributed (approximately) in the same proportion as in *SCT*. The reason for not guaranteeing requirement (c) is that there is no control over classes from other sub-hierarchies that are added and become new target nodes when following the right-hand expressions of the seeds.

Therefore, in order not to conflict with requirements (a) and (b) when creating *M*, the only possibility is to carefully select the initial signature that bootstraps the modularization algorithm. For that purpose, we investigated an iterative algorithm that dynamically adjusts the distribution of classes used as seeds in the initial signature. Before presenting the algorithm, we introduce the following notation:

- As introduced before, *SCT* represents the OWL EL version of SNOMED CT used as input. Sub-hierarchies are termed *SH*_*k*_.
- *M* represents the output module, whose sub-hierarchy distribution should match *SCT*’s as closely as possible (Table 1).
- *SIGN* is the input signature, consisting of classes from *SCT*, that is used to bootstrap the modularization process described in Subsection ‘Module construction from seeds’.
- $Error(SH_{k})=Size(M_{SH_{k}})-Size(SCT_{SH_{k}})\phantom {\dot {i}\!}$ indicates the error on a per sub-hierarchy basis. Errors are calculated in percentage terms (see distribution in Table 1).
- $RSS = \frac {1}{18}\sum _{k=1}^{18}Error(SH_{k})^{2}\phantom {\dot {i}\!}$, where *RSS* represents the residual sum of squares. Convergence of the algorithm is defined when *RSS*<1.

The algorithm, at each iteration *i* is the following:

1. A random signature *SIGN*_*i*_ consisting of 2000 classes from *SCT* is selected, following the same class sub-hierarchy distribution as *SCT*, and ensuring that all sub-hierarchies in the signature contains at least one class.
2. A module *M*_*i*_ is computed following the principles described in Subsection ‘Module construction from seeds’. Its sub-hierarchy distribution is calculated.
3. Convergence is checked. If *RSS*>=1, Steps 1 to 3 are repeated after adjusting the scaling factor for the sub-hierarchy distribution of the signatures in the next iteration *i*+1:
  $f\left (SIGN_{i+1_{SH_{k}}}\right) = f\left (SIGN_{i_{SH_{k}}}\right) \times \frac {f\left (SCT_{SH_{k}}\right)}{f\left (M_{i_{SH_{k}}}\right)}$ with $f\!\left (\!M_{i_{SH_k}}\!\right)$ being the relative frequency of sub-hierarchy *SH*_*k*_ measured in the resulting module in iteration *i*, *M*_*i*_.

## Results

**Fig. 4:**
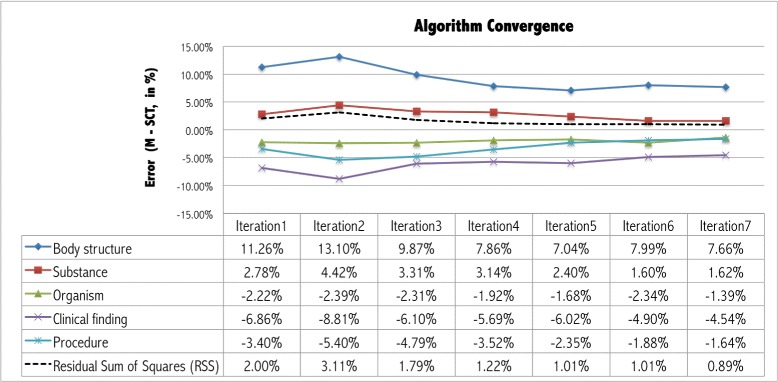
Execution of the algorithm, showing convergence in iteration 7. Each line represents the difference in distribution for a particular sub-hierarchy of a balanced module at a given iteration, when compared to SNOMED CT. For example, in the balanced module at iteration 1, *Body Structure* is proportionally 11.26 % bigger than in SNOMED CT (it is over-represented), while *Clinical Finding* is 6.86 % (it is under-represented). The *dashed* line represents the residual sum of squares of all sub-hierarchies, 0 meaning that the sub-hierarchies in the balanced module are distributed in exactly the same way as in SNOMED CT (perfectly balanced module)

A module *M* with 10 834 classes was extracted from 2000 seeds, the module being in 4.7 % the size of the original *SCT* (229 330 classes). Figure 4 shows how the algorithm converged after 7 iterations, the error for sub-hierarchies exceeding an error of 1 %, and the residual sum of squares.

**Fig. 5:**
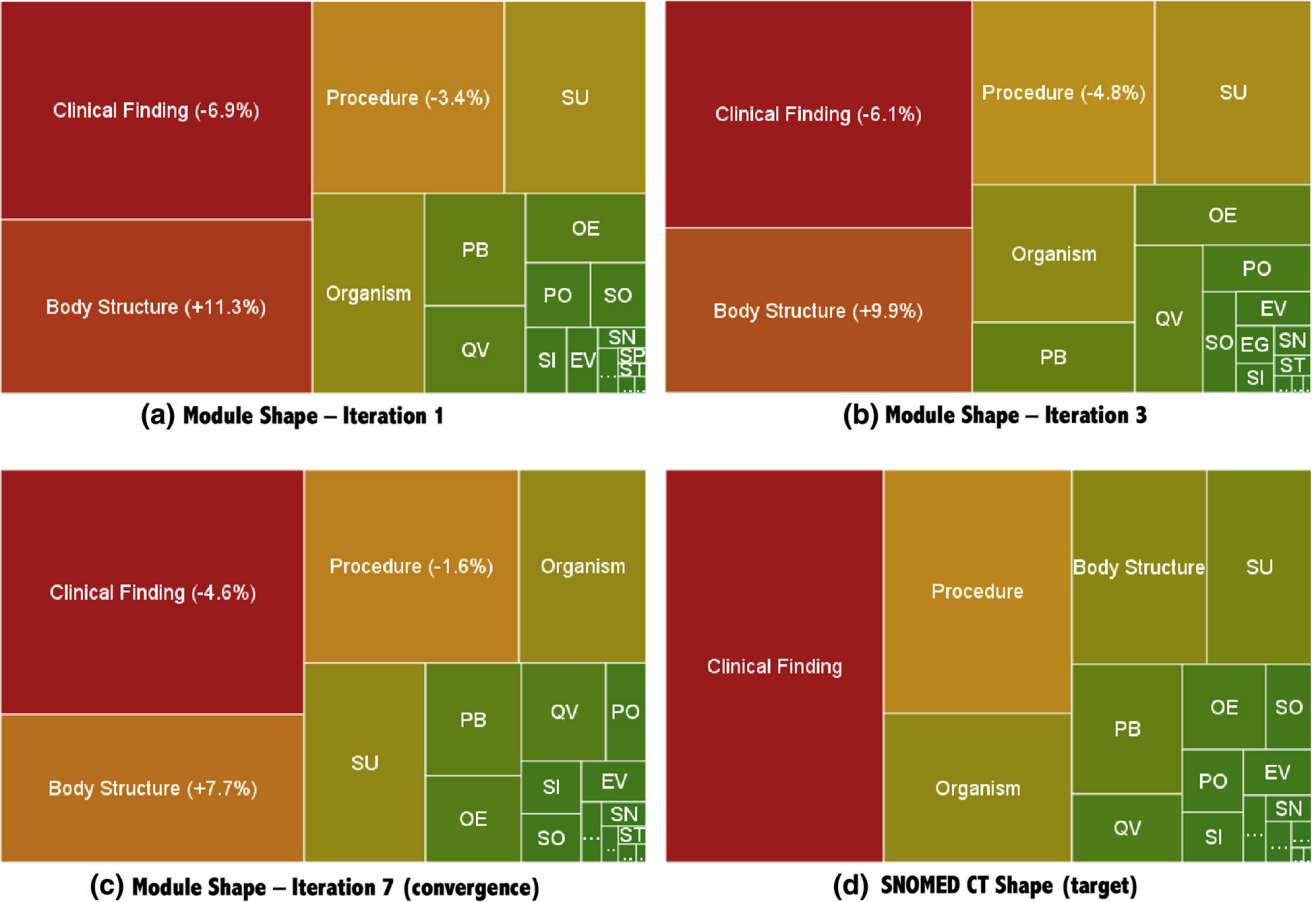
Visual comparison of the shape between modules *M* and SNOMED CT (**d**) in iterations 1 (**a**), 3 (**b**), and 7 (convergence, **c**). Clinical Finding, Procedure, and Organism were under-represented, while Body Structure and Substance were over-represented

As can be seen in the table below the graph, the sub-hierarchies *Clinical Finding*, *Procedure*, and *Organism* were under-represented in *M*, while *Body Structure* and *Substance* were over-represented. The same results can be confirmed graphically in the treemaps shown in Fig. 5, at iterations 1, 3, and 7.

These results were partly expected, due to the nature of the modularization approach that uncontrollably adds extra classes that appear in right-hand expressions to preserve SNOMED CT’s class definitions. The most representative example is the sub-hierarchy *Body Structure*, whose concepts appear often in definitions in *Clinical Findings*, e.g. *‘Finding site: Bone structure of femur (body structure)’* for *‘Fracture of femur (clinical finding)’*.

Our experience indicates that there is a point (around 7 iterations) where the algorithm starts oscillating, and the residual sum of squares can not diminish any longer. In practice, this means that when the algorithm tries to compensate the under-representation of *Clinical Finding* by adding more *Clinical Finding* seed concepts to the signature in the next iteration, the result of the new balanced module inevitably includes also more *Body Structure* concepts. This is better understood using Fig. 3 as an example. Assuming concept 10 is a *Clinical Finding* seed added to compensate their under-representation, the graph-traversal modularization algorithm would also add *Body Structure* concepts 17, 16, 15, and 9 to the balanced module, because concept 17 appears in a right-hand expression of concept 10, and 16, 15, and 9 are its ancestors (Fig. 2).

## Discussion

Our results suggest that it is difficult for ontology modules to meet all of our modularization criteria without relaxing the constraints of how concepts in the modules are distributed by sub-hierarchies: this is because modularization criteria are conflicting. In our experiments, all obtained modules over-represented or under-represented some of SNOMED CT’s sub-hierarchies to varying degrees.

The error figures that we obtained after convergence, however, never reached 8 % for any sub-hierarchy and all our modules contained a fair representation of every sub-hierarchy. Furthermore, convergence was reached after only 7 iterations and the resulting module was 4.7 % the size SNOMED CT. Such modules might be sufficient in many of the use cases that motivated their creation, i.e., extracting modules that show an (approximate) concept distribution to the one shown in SNOMED CT.

In this study, we focused on extracting balanced modules in SNOMED CT only, both for practical purposes (useful input for related SNOMED CT research) and because its size and complexity make SNOMED CT an excellent case study. Our approach, however, should work similarly with for any ontology where graph-traversal modularization techniques based on an input signature apply. The literature currently reports positive experiments with NCI, GALEN, GO, SUMO, SWEET, and DOLCE-Lite [8, 13].

## Conclusions

Modules that preserve the concept distribution by sub-hierarchy of the original ontology have been generally neglected in the field of ontology modularization. However, balanced modules are extremely useful in applications such as ontology-based quality assurance, scaling experiments for real-time performance, or when developing scalable testbeds for software tools.

In this study, we have proposed and evaluated an iterative algorithm to investigate to what extent extracting such balanced modules in SNOMED CT is possible. Our results show that graph-traversal ontology modularization techniques relying on an input signature can indeed be used, if: the process is repeated a number of times; the input signature is dynamically adjusted in each iteration; and a moderate under/over-representation of some hierarchies is tolerated.

Several questions are still open and need to be addressed as future work: how to select a minimal signature; how signature size influences the final size of the modules; and how a change in the randomization process of the signature selection (e.g., by stratifying the randomization by node depth) influences the concept distribution of the module. In addition, a validation of our experiments using other ontologies and comparing the results would provide a more comprehensive overview.

Our results, however, conclusively show that SNOMED CT can be squeezed to less than 5 % its size without any sub-hierarchy losing its shape more than 8 %, which is likely to be sufficient in most use cases.
